# Risk of dysphonia and voice quality in performing arts students

**DOI:** 10.1590/2317-1782/20232022036en

**Published:** 2023-08-21

**Authors:** Eloisa Pinheiro Ferrari, Marcia Simões-Zenari, Suely Master, Katia Nemr

**Affiliations:** 1 Faculdade de Medicina, Universidade de São Paulo - USP - São Paulo (SP), Brasil.; 2 Instituto de Artes, Universidade Estadual Paulista de Júlio Mesquita Filho - UNESP - São Paulo (SP), Brasil.

**Keywords:** Voice, Voice Quality, Voice Disorders, Dysphonia, Art

## Abstract

**Purpose:**

To analyse the relationship between the risk of dysphonia and vocal quality in undergraduate performing arts students.

**Methods:**

Observational cross-sectional study with 38 undergraduate students in Performing Arts. We applied screening protocols for general and specific risk of dysphonia for actors and made recordings of sustained emission of the vowel /a/, spontaneous speech and reading a text, used for perceptual analysis performed by three evaluators using the GRBASI scale. After intra and inter-rater reliability tests it was obtained final classification of the general degree of vocal deviation parameter for each participant. Comparisons were made considering groups that had or did not have other profession/activity with the use of voice, and the groups were formed from the general grade.

**Results:**

Most students were at high risk for dysphonia. All had vocal alteration, with a predominance of mild degree. Students who had another profession/activity with voice use scored higher in the specific protocol for actors, and in the sum of this protocol with the general screening protocol. There was no relationship between the degree of vocal alteration and the risk of dysphonia. Students who did not yet work professionally had more moderate or severe vocal alterations, and those who did work professionally had a higher frequency of mild vocal alterations.

**Conclusion:**

Most students were at high risk for dysphonia. All had vocal alteration, with a predominance of mild alteration. There was no relationship between the risk of dysphonia and the degree of vocal alteration.

## INTRODUCTION

Voice is a relevant element of speech therapy work with actors for encompassing the field of expressiveness and vocal resources, aiming to research and understand all organs involved in voice production to meet the needs of stage voice without wearing it out^(1,2)^.

Vocal quality, as well as clear and accurate articulation and pronunciation, are fundamental for actors to guarantee the intelligibility of their performance. Articulatory and vocal distortions are tolerated only when linked to the stage creative process^(3)^. The literature reports studies demonstrating that actors are a risk group for the onset of voice disorders, with alteration and damage in vocal quality^(4,5)^.

An actor’s voice might also be influenced by factors like inadequate environmental, acoustic, and climatic conditions, as well as general health and psychological issues^(6)^. Potential voice problems in actors emerge from the lack of mastery of the vocal tool, in addition to body tensions, including in the speech apparatus, pneumo-phono-articulatory incoordination, and the presence of inadequate habits since the training period^(7,8)^.

Only a few studies analyze the students’ voice, either in drama courses or in the Performing Arts undergraduate course ^(9)^. However, one study found the presence of vocal and laryngeal alterations in half of the drama students evaluated^(10)^. Another research investigated the prevalence of vocal disorders and the knowledge of vocal health and alteration during the training period and the first five years after the undergraduate course. Professional actors mentioned a higher occurrence of vocal alterations in the five first years after the training and learned less about vocal health than those from classical singing and musical drama^(11)^.

It is important that the training of actors cover voice training techniques and lessons on vocal health and well-being, in addition to associating vocal care with the understanding of vocal anatomy and physiology. Gathering a wide and deep set of information on the features of voice and lifestyle of these students might help teachers of vocal techniques and speech therapists who act in this area use such information as a reference to evaluate the vocal behavior of students, in addition to identifying which areas need greater assistance. Students might also better understand their conduct and develop vocal self-perception as a response to habits and behaviors^(12)^.

To obtain and deeper investigate information related to vocal aspects, studies generally use the initial analysis, or anamneses, since the subject reflects and provides relevant data to compose the assessment and speech therapy planning^(13)^. The collection can be performed through the Screening Protocol of Risk of Dysphonia - General (SPRD-G)^(14)^, a questionnaire that scans the risk of dysphonia in individuals of any age and gender through quantitative and qualitative analyses. This tool is a robust instrument for the screening, guiding, and prevention of voice disorders, in addition to benefiting treatment planning. Specific complementary protocols to the SPRD-G have been developed, such as the Screening Protocol of Risk of Dysphonia in Actors (SPRD-A)^(15)^, aiming to investigate the influence of work environment and organizational factors on acting.

The analysis of voice quality and measure of the risk of dysphonia enlarge the knowledge of vocal features and gather information on the use of voice in acting, lifestyle, vocal health, and vocal self-perception of the Performing Arts students. These data might contribute to creating actions and strategies to improve training and favor the students’ self-awareness concerning vocal habits, and behaviors, and use for them to adopt care and practices target to their routines and needs.

This study aimed to analyze the relationship between the risk of dysphonia and vocal quality in the students of an undergraduate course in Performing Arts. We also aimed to compare the risk of dysphonia and vocal quality between the students who, in addition to acting, had another occupation or activity using the voice, and those who practiced only acting.

## METHODS

### Type of study

This is a transversal observational study approved by the Ethics Committee for the Analysis of Research Projects of the Hospital das Clínicas, at the Medicine School of the University of São Paulo, number (n° 3.315.275), approved by the coparticipant institution.

### Participants

All students from all terms of the Undergraduate Course in Performing Arts at a higher education institution enrolled in 2019 were invited to participate in the research. Those individuals who had any involvement or diagnosis that limited communication or the performance of the proposed tasks were disqualified. The sample was composed of 38 students, from the first to the last term of the course, including 11 males and 27 females, with a mean age of 21.3 years (dp=2.50), and a median of 20.5, varying between 18 and 29 years old. All signed the Free and Informed Consent Form (ICF).

### Procedures

#### Protocols

All participants filled in the following two instruments:

General Dysphonia Risk Screening Protocol (G-DRSP)^(14)^, composed of initial questions of personal identification and 18 other questions divided into the following subitems: history of dysphonia, signs, and symptoms, use of voice outside work, nutrition, hydration, medications, contact with smokers, sleep, history of diseases, family history of vocal disorders, physical activities, and leisure, among others. It also encompasses vocal self-evaluation based on a Visual Analog Scale (VAS), which is measured through a millimeter ruler, with values between 0 (no disorder) and 10 (maximum disorder). The calculation of the total score of each subitem generates a partial score, which is then added to the value obtained from the vocal self-evaluation. This sum results in a total maximum score of 131 points, and the higher the final score, the higher the risk of dysphonia. The cutoff reference scores for high-risk dysphonia are 29.25 for female individuals and 22.75 for male individuals^(14)^.

Dysphonia Risk Screening Protocol - Actors (DRSP-Actors)^(15)^, composed of 24 questions divided into subitems, such as training, singing lessons, mean time of voice use, vocal involvement, the practice of vocal warm-up and cool-down, rehearsals/lessons, environmental conditions for the use of voice in acting, respiratory type, vocal psychodynamics, voice perception, having other occupations or activities that use the voice, consumption of smoking, alcohol, and drugs, use of dental prosthesis, and questions for women on pre-menstrual symptoms, pregnancy, menopause, and hormonal alterations. The score is formed from the sum of the points of each subitem. The total score might vary from 0 to 100, and the higher the score, the higher the risk of dysphonia. The cutoff values for the risk of dysphonia for this protocol are yet to be defined.

Following the application and measurement of the SPRD-G total score, the participants were divided into two groups according to the risk classification: high and low risk of dysphonia based on the cutoff point previously defined by the protocol, according to gender. The SPRD-A total score was also calculated and the sum of the total of the protocols generated the final score (SPRD-Final). This score allows quantifying the risk of dysphonia considering general factors, to which the whole population is exposed, and specific factors of the occupation, herein the agents related to personal factors, organization, and acting environment.

### Voice recording

The speech samples were collected using iPad*®* (MP2F2BZ/A, iOS 10.3.3), Shure Motiv*®* application developed by Shure*®*, at 44.100 Hz, monosound in WAV format, with an attached unidirectional microphone, MOTIV MV88*®* model (Shure, United States), positioned at 15 cm from the participant’s mouth, at an angle of 45°. Such a measure was adopted for presenting a better quality after the tests using the microphone at different positions and distances. In the same acoustically controlled environment, with noise below 50 dB, through the SoundMeter application, developed by Digital SoundMeter*,* each participant was positioned sitting down for the recording of the following tasks: sustained vowel emission /a/ for three to five seconds, three times, applying usual pitch and loudness; spontaneous speech of the question: “Talk about the importance of voice for the actor”; and reading the text “The discovery of Cristóvão Colombo”, by Mowa Lebesque^(2)^. The researcher held the paper showing the text for the participants to read and instructed them to start with a silent reading to familiarize themselves with the text and then read it in their usual tone.

### Auditory perception of speech

The auditory perception of speech was conducted by three examiners. They are speech therapists and voice specialists with the time of experience between eight and fifteen years, in addition to being familiar with the assessment scale and having practice with professional voice. All voice recordings were stored with 20% of random repetition for intra-examiner reliability analysis.

Each examiner conducted an auditory perception of speech analysis independently using the GRBASI scale. They were instructed to evaluate the sample of the vowel /a/ emission first, then, after a week, the spontaneous speech and text reading analyses to help reduce the impact on the evaluator’s memory. The five sentences of the first half of the text were extracted and read by all participants more fluently. The examiners could hear each recording track as many times as they needed for the analyses. For purposes of analysis, this research considered only the G (general degree of vocal deviation) of the assessments of the tasks.

The examiners were calibrated through anchor samples of the voice features and/or mild deviation degree. The ideal number and type of anchor are unknown; therefore, we used 16 anchor stimuli for the sustained vowel /a/ emission and sentences from the CAPE-V protocol, or spontaneous speech samples containing four samples of vocally healthy individuals, four samples of individuals with mild vocal deviation, four samples of individuals with from mild to moderate vocal deviation, and four samples of individuals with intense vocal deviation. Each degree of vocal deviation contained two samples of male voices and two samples of female voices. The examiners were instructed to hear the anchor stimuli immediately before the analysis of the participants’ voices.

### Data analysis

The descriptive analysis encompassed absolute and relative frequencies and the respective measures of central trend and dispersion.

The intra- and inter-evaluator analyses of auditory perception of speech were calculated using the Intraclass Correlation Coefficient (ICC). The statistical significance value of 5% (*p* ≤ 0.05) and a confidence interval of 95% were adopted. The following classification was established: values below 0.5 - poor reliability; between 0.5 and 0.75 - moderate; between 0.75 and 0.90 - good, and above 0.90 - excellent^(16)^. After the reliability analyses, we used the data corresponding to the examiners with higher intra- and inter-evaluator correlation indices in at least one of the assessments: vowel/a/, spontaneous speech, and text reading.

Examiners 1 and 2 presented a moderate agreement (0.53; *p* < 0.001) concerning the vowel /a/ task, according to the analysis of inter-evaluators' agreement. For the same task, the intra-evaluator reliability for these examiners indicated an excellent (1.0; *p*< 0.001) and moderate (0.71; *p* < 0.019) reliability, respectively. Thus, we selected the analyses of evaluators 1 and 2 for the vowel /a/ task to establish the final classification of G for each participant. The mean of the assessments was adopted in the case no agreement was reached between the evaluators. The samples were then ranked based on the following classification: 0 to 0.4 - normal variation of voice quality (0); 0.5 to 1.4 - mild variation (1); 1.5 to 2.4 - moderate variation (2), and 2.5 to 3.0 - severe variation (3)^(17)^. According to this classification, the participants were divided into two groups: mild vocal alteration degrees (degree 1); and moderate and severe vocal alteration degrees (degrees 2 and 3).

The normality and homoscedasticity assumptions were verified through the Shapiro-Wilk and Mildne tests, respectively, defining the exact Fisher test (data category), Student t-test (quantitative data, for unfulfilled normality assumption), and Mann-Whitney U test (quantitative data, for unfulfilled normality assumption). If the heteroscedasticity assumption was unfulfilled, the *p*-value was calculated through Welch’s correction. In addition, we considered the *d* (parametric) or *r* (non-parametric) coefficients, when appropriate, to verify the size of the effect of the difference between the groups. The criteria adopted for the *d* coefficient were small - between |0.200| and |0.499|; medium - between |0.500| and |0.799|, and big - above |0.800|. In turn, the *r* coefficient was considered small - between |0.100| and |0.299|, medium - between |0.300| and |0.500|, and big - above |0.500|.

The following comparisons were established:

- Subitem another occupation/activity using the voice (OP), comparison of the groups of individuals who had another occupation and those who did not concern the total score of the SPRD-G; vocal self-evaluation subitem (VSE); total score does SPRD-A; SPRD-Final; professional acting and vocal instructions (questions of the training subitem); and risk of dysphonia;
- Degree of vocal alteration, groups with mild alteration and moderate/severe alteration were compared concerning the total score of the SPRD-G; vocal self-evaluation subitem (EAV); total score of the SPRD-A; another occupation/activity subitem using the voice (OP); professional vocal acting and instructions (questions of the training subitem); SPRD-Final, and risk of dysphonia.

The inferential analyses adopted a statistical significance value of 5% (*p* ≤ 0.05). The confidence intervals of 95% were calculated using the bias method corrected and accelerated based on 2000 bootstrap samples. All analyses were performed on the SPSS^®^ statistical software (Statistical package for the social sciences), version 25.0.

## RESULTS

The means of the total sample were 37.62 (±8.81) for the SPRD-G, 26.18 (±6.93) for the SPRD-A, and 64.00 (±12.23) for the SPRD-Final. As to the classification of risk of dysphonia, four participants (10.5%) presented low risk and 34 participants (89.5%) showed high risk.

Based on the G parameter, the auditory perception of speech analysis showed means of 1.37 (±0.49) and 1.16 (±0.79), obtained by evaluators 1 and 2, respectively, for the /a/ vowel task, while the spontaneous speech and text tasks reached 1.05 (±0.46) and 0.63 (±0.71), respectively. The division of groups according to the degree of vocal alteration resulted in 22 participants (57.9%) with mild vocal alteration and 16 participants (42.1%) with moderate or severe vocal alteration.

As to the comparisons, a statistically significant difference occurred in the total scores of the SPRD-A and the SPRD-Final, where individuals who had another occupation or activity using the voice scored more than those who had not (Table 1).

**Table 1:** Descriptive values and comparative analysis of the individuals who had and did not have another occupation/activity using the voice concerning the scores of the SPRD-G, SPRD-A, SPRD-Final, and EAV instruments

**Instruments**	**Other occupations/activities using the voice**	**n**	**Mean**	**SD**	**Median**	**Min.**	**Max.**	***p***	**E.S.**
SPRD-G	No	23	36.13 [33.37. 38.94]	7.36	37.00 [33.00. 39.00]	22.00	51.00	0.308^a^	0.408^d^
	Yes	15	39.13 [33.93. 43.87]	10.59	41.00 [37.00. 44.00]	15.00	56.00		
SPRD-A	No	23	22.91 [20.87. 25.04]	5.05	23.00 [20.00. 25.00]	15.00	34.00	**<0.001^a^** *	1.640^d^
	Yes	15	31.20 [28.40. 34.27]	6.50	28.00 [26.00. 36.00]	25.00	43.00		
SPRD-Final	No	23	59.13 [55.89. 62.61]	9.57	57.00 [56.50. 58.00]	37.00	86.00	0.004^a^*	1.170^d^
	Yes	15	70.33 [64.20. 75.80]	12.59	73.00 [68.00. 73.00]	40.00	90.00		
EAV	No	23	4.00 [3.61. 4.41]	1.09	4.00 [4.00. 4.00]	2.00	6.50	0.718^b^	0.061^r^
	Yes	15	3.87 [3.07. 4.60]	1.55	4.00 [3.00. 5.86]	0.00	7.00		

The student t-test for independent samples (^a^) and Mann-Whitney U Test (^b^)

* Statistically significant Value at the level of 5% (*p* ≤ 0.05)

d Parametric coefficient to verify the effect size of the difference between groups

r Non-parametric coefficient to verify the effect size of the difference between groups

**Caption:** VAS = Visual Analog Scale; SD = Standard Deviation; Min. = Minimum; Max. = Maximum; E.S. = Effect Size

There was no statistically significant difference between having or not another occupation using the voice concerning the analyzed variables: risk of dysphonia, professional acting, and vocal instructions (Table 2).

**Table 2:** Comparison between those who had another occupation/activity using the voice concerning the risk of dysphonia, professional acting, and vocal instructions

**Variables**	**Conclusion**	**Other occupations/activities using the voice**						***p***
		No		Yes		Total		
		n	%	n	%	n	%	
Risk of dysphonia	Low	3	13.0	1	6.7	4	10.5	> 0.999
	High	20	87.0	14	93.3	34	89.5	
Professional acting	No	11	47.9	6	40.0	17	44.7	0.744
	Yes	12	52.1	9	60.0	21	55.3	
Vocal instructions	No	13	56.5	10	66.7	23	60.5	0.736
	Yes	10	43.5	5	33.3	15	39.5	

Fisher’s exact test

All individuals with a mild degree of alteration and those with moderate or severe degrees of alteration showed similar results in the scores of the SPRD-G, SPRD-A, SPRD-Final, and EAV (Table 3).

**Table 3:** Descriptive values and comparative analysis of the degrees of vocal alteration concerning the scores of the SPRD-G, SPRD-A, SPRD-Final. and EAV instruments

**Instruments**	**Degree**	**n**	**Mean**	**SD**	**Median**	**Min.**	**Max.**	***p***	**E.S.**
SPRD-G	Mild	22	37.07 [32.82. 41.36]	10.47	37.50 [31.50. 42.75]	15.00	56.00	0.828^a^^w^	0.056^d^
	Moderate or severe	16	37.66 [34.90. 40.31]	5.98	38.50 [35.00. 40.00]	27.00	47.00		
SPRD-A	Mild	22	25.82 [23.40. 28.36]	6.22	25.00 [24.50. 27.00]	15.00	41.00	0.708^a^	0.140^d^
	Moderate or severe	16	26.69 [23.06. 30.31]	8.00	25.00 [21.50. 28.00]	15.00	43.00		
SPRD-Final	Mild	22	62.93 [57.52. 68.39]	13.44	64.50 [55.50. 69.00]	37.00	86.00	0.798^b^	0.043^r^
	Moderate or severe	16	64.41 [60.03. 69.06]	10.19	63.50 [57.50. 66.00]	53.50	90.00		
EAV	Mild	22	3.93 [3.41. 4.45]	1.32	4.00 [4.00. 4.00]	0.00	6.50	0.686^b^	0.068^r^
	Moderate or severe	16	3.97 [3.44. 4.59]	1.24	4.00 [3.00. 4.00]	2.00	7.00		

The student t-test for independent samples (^a^) and Mann-Whitney U Test (^b^)

d Parametric coefficient to verify the effect size of the difference between groups

r Non-parametric coefficient to verify the effect size of the difference between groups

w Welch’s correction of heteroscedasticity

**Caption:** VAS = Visual Analog Scale; SD = Standard Deviation; Min. = Minimum; Max. = Maximum; E.S. = Effect Size

We found a statistically significant difference between the degree of vocal alteration and professional acting. The students who had not acted professionally presented greater vocal alteration of moderate or severe degrees, while those who acted professionally showed a mild degree of alteration (Table 4).

**Table 4:** Comparison of the degrees of vocal alteration concerning the risk of dysphonia, other occupations/activities using the voice, professional acting, and vocal instructions

**Variables**	**Conclusion**	**Degree of vocal alteration**						***p***
		Mild		Moderate or severe		Total		
		n	%	n	%	n	%	
Risk of dysphonia	Low	4	18.18	0	0.00	4	10.53	0.124
	High	18	81.82	16	100	34	89.47	
Other occupation or activity using the voice	N	14	63.64	9	56.25	23	60.53	0.743
	Yes	8	36.36	7	43.75	15	39.47	
Professional acting	No	6	27.27	11	68.75	17	44.74	**0.020** *
	Yes	16	72.73	5	31.25	21	55.26	
Vocal instructions	No	12	54.55	11	68.75	23	60.53	0.506
	Yes	10	45.45	5	31.25	15	39.47	

Fisher’s exact test

* Statistically significant value at the level of 5% (*p* ≤ 0.05)

## DISCUSSION

This study analyzed the relationship between the risk of dysphonia and vocal quality in students of the Performing Arts undergraduate course due to the lack of research in the literature addressing this type of information jointly in this population. Some studies explore the voice features of Performing Arts students^(10)^ and others approach information on relevant variables of vocal health^(13)^.

According to the results of the screening protocols for risk of dysphonia from the SPRD-G, based on the analysis of general factors related to vocal alteration, such as health, lifestyle, and habits, the mean obtained was superior to the cutoff values for high-risk dysphonia. The use of this instrument was also effective in identifying and ranking the risk of dysphonia in the population in general^(14)^, as well as in professional actors ^(15)^, musical drama actors^(18)^, and teachers^(19)^.

The study involving professional actors showed a predominance of high risk of dysphonia^(15)^, thus corroborating our research with students. Thereby, we may assume that the risk of dysphonia might be present in actors since the training period. For this category, previous studies found a high prevalence of vocal alterations^(10, 20)^. Another research applied a specific form for the collection and register of information on Performing Arts students, showing reports of a series of vocal behaviors and lifestyles that might increase the risk of vocal alteration^(13)^.

Complementarily to the general protocol, specific screening instruments for the risk of dysphonia have also been developed, such as for children^(21)^ and voice professionals, like musical drama actors and teachers^(18,19)^. Specific protocols for voice professionals have proved to be useful tools for qualitative analyses for not differentiating professionals with and without vocal alteration since both are exposed to the same risks. However, these protocols provide relevant specific data related to the professional activity that might influence the risk of dysphonia^(15,19)^. Other proposals for analysis of voice-related factors in actors have been developed; however, the approach of these aspects in the protocol in our study and those found in the literature had no similarity^(6,9,22)^.

Regarding the auditory perception analysis of vocal quality, despite not being the focus of this research, the means obtained in the general degree of vocal deviation were higher in the vowel assessment than in the other tasks, considering the analyses of the selected examiners. In general, vocal alteration is more noticeable in the assessment of sustained vowel emission than in linked speech tasks^(23)^.

The final classification of G showed the presence of vocal alterations in all participants, predominantly mild degrees; however, it is worth noting that many of them presented moderate or severe degrees. Such findings do not corroborate other research involving professional and amateur actors or drama students who performed the auditory perception analysis and reached compatible means with vocal quality without alteration^(4,15,24,25)^. For a better characterization of the vocal alterations observed, we suggest deeper analyses of other aspects of the GRBASI scale. Likewise, a greater auditory perception of speech, also considering the glottal source filter, and other analyses, such as acoustics, should also be considered.

Regarding the presence of a vocal alteration in the entire sample, a potential hypothesis is that the students who accepted to participate in the study had noticed the presence of vocal alteration, even though the vocal self-evaluation, subitem to the SPRD-G, was low. In general, the students did not perceive a great level of alteration in their voices. In addition, as discussed later, there was no relationship between the vocal self-evaluation and the degree of vocal alteration.

In the comparison between individuals who had another occupation/activity using the voice and those who did not, the former reached the higher scores in the SPRD-A protocol, hence higher values in the sum of this protocol and the SPRD-G, the SPRD-Final. The value generated from the underscore related to voice use in other activities had a great weight in the sum of the protocol total score. Thus, such a result demonstrates that this aspect is a relevant factor in the SPRD-A score to differentiate the students.

Like in our research, studies involving actors commonly observe the use of voice in another occupation or activity other than acting^(6,22)^. This scenario might be linked to the difficulty of many actors to support financially by dedicating exclusively to acting, often because of the instability of the work market and lack of appreciation. In the case of the studied population, because the participants are students, it is even clearer the need for other activities or occupations for financial support. In addition, these students are inserted in a training environment where several complementary practices are available and might use their voices for other demands, especially those related to singing. Thereby, considering that the literature points out a relationship between the presence of complaints and high vocal demand, including from the use of voice in other activities, we suggest that such an aspect is highlighted and targeted in further studies involving this population^(8)^.

Finally, as to the comparisons based on the degree of vocal alteration, the students who were not acting professionally presented more moderate or severe degrees of vocal alterations, while those who acted professionally presented predominantly mild degrees of alteration; these findings do not agree with the literature^(9,24)^. The time of professional acting was not investigated in this study. The literature reports a mean of between five and 20 years of experience^(4,8,13)^. Professional acting, regardless of time, might be a preventive factor for vocal alteration in this studied sample. Further studies should investigate its relationship with other factors, such as the practice of warm-up and cooling-down exercises, vocal training, and the presence of certain habits and lifestyles, among other aspects that might help prevent vocal alterations.

We also highlight that no relationship was found between the risk of dysphonia and the degree of vocal alteration, demonstrating that the high risk of dysphonia did not generate a higher degree of vocal alteration. Previous studies with professional actors^(15)^ and teachers^(19)^ found the same results. The consulted literature reported no studies addressing such a relationship with drama/Performing Arts students.

Research covering larger samples of both professional and student actors should analyze deeper the aspects that might be related to vocal alteration, seeking more accurate approaches for the prevention and promotion of vocal health in these individuals. Most studies with actors addressing the risk of dysphonia and vocal quality conducted comparative analyses between the pre- and post-drama performances. The findings vary from improvement or maintenance of vocal quality to the presence of risk of dysphonia^(4,25)^. Concerning the students, one study compared vocal quality, anamneses information, and self-evaluation of groups with and without laryngeal alteration, and found better results for the second group^(10)^.

Additionally, since there was no relationship between the vocal self-evaluation and the degree of vocal alteration, the participants evaluated their voices more positively than the speech therapists did. However, it is worth considering that the examiners in this research evaluated some aspects of voice production through specific tasks, especially the isolated vowel emission. The students do not have the same technical knowledge, hence, by self-evaluating, they consider as reference their wider vocal use on a day-to-day basis.

The literature also reports a study that found a disagreement between the self-perception of vocal quality by professional actors and other subjects^(9)^. The authors described the relevance of actors being able to identify vocal alterations more accurately for a more prompt search for specific treatments, thus softening the risk of complications and consequent restricted voice use in acting performance.

Thereby, it is important to work with actors’ vocal perception since the training period to favor the identification of vocal alterations evidence. Thereby, vocal resources might favor the freedom of interpretation. In addition, vocal self-evaluation is associated with auditory perception and studies have found that behavioral dysphonia might be related to disorders of auditory processing^(26,27)^.

Regarding the absence of association between vocal instructions and the degree of vocal alteration, the literature reports no differences concerning the vocal knowledge of students with and without vocal complaints^(8)^. Another study found that the knowledge and practice of vocal well-being did not influence the vocal assessments performed by the actors pre- and post-drama performance^(5)^.

The sample encompassed students at the start of their training process who might not have received instructions about vocal well-being yet. Even the students who had received them might not have applied the information since often actors are more concerned with mastering the character than with vocal health^(6)^. Thereby, in addition to the knowledge on vocal well-being, it is relevant to improve approaches that address the enhancement of actor’s body-vocal-emotional performance for the maintenance of vocal health, especially in drama courses and Performing Arts undergraduate courses, for actors to be informed adequately and adhere to the practices even in the training process.

The vocal quality assessment and measurement of the risk of dysphonia provided knowledge on the vocal features, in addition to information regarding acting-related aspects lifestyle, and behaviors. The use of the screening protocols for the risk of dysphonia might have favored the reflection and self-awareness of students concerning their habits and behaviors, promoting engagement and increasing adherence to care and adoption of healthier habits, in addition to a more aware vocal use.

Concerning the limitations of this study, a complementary assessment of speech therapy would be relevant, as well as referring the participants to an otorhinolaryngological evaluation since the literature reports that actors generally present laryngeal alterations due to behavioral dysphonia^(28,29)^.

Multivariate and deeper analyses of other data from screening protocols for the risk of dysphonia might also be relevant for further studies for greater details and understanding in addition to our findings.

Vocal assessment is a set of procedures to identify and characterize vocal behavior, vocal quality, and adjustments for voice production. Therefore, the more complete, the better the understanding of the relationship between the risk of dysphonia and vocal quality. Based on individual features of each student/artist's voice, their vocal habits, and behavior, targeted training can be proposed for the development and specific improvement of each voice, preserving health and enhancing the actor’s body-vocal-emotional performance^(29)^.

Furthermore, it is worth considering the analysis of the central auditory processing in this population since difficulties in auditory skills interfere with the adequate follow-up of vocal production, which might favor the maintenance of inadequate vocal standards and influence the perception of voice modulation^(30)^ - a fundamental factor for stage activities.

## CONCLUSION

Our sample of students from the Performing Arts undergraduate course revealed that most of them presented a high risk of developing dysphonia and all showed some vocal alteration, although predominantly to a mild degree.

Those students who had another occupation or activity using the voice reached higher scores in both the SPRD-A and the SPRD-Final.

The students who had not acted professionally presented moderate or severe degrees of vocal alteration more frequently, while those who acted professionally showed a mild degree of alteration. There was no relationship between the degree of vocal alteration and risk of dysphonia.
