# Supplementary material for: Risk of dysphonia and voice quality in performing arts students
Source: Codas. 2023 Aug 21;35(4):e20220036. doi: 10.1590/2317-1782/20232022036en (PMC10446748; doi:10.1590/2317-1782/20232022036en)
Supplement: Translation

# Translation: Risco de disfonia e qualidade vocal em estudantes de artes cênicas

**DOI:** 10.1590/2317-1782/20232022036pt

**Authors:** Eloisa Pinheiro Ferrari, Marcia Simões-Zenari, Suely Master, Katia Nemr

**Affiliations:** 1 Faculdade de Medicina, Universidade de São Paulo - USP - São Paulo (SP), Brasil.; 2 Instituto de Artes, Universidade Estadual Paulista de Júlio Mesquita Filho - UNESP - São Paulo (SP), Brasil.

**Keywords:** Voz, Qualidade da Voz, Distúrbios da Voz, Disfonia, Arte

## Abstract

**Objetivo:**

Analisar a relação entre o risco de disfonia e a qualidade vocal em estudantes de graduação em Artes Cênicas.

**Método:**

Estudo transversal observacional com 38 estudantes de graduação em Artes Cênicas. Foram aplicados protocolos de rastreio de risco de disfonia geral e específico para atores, e realizadas gravações da emissão sustentada da vogal /a/, fala espontânea e leitura de texto, usadas para análise perceptivo-auditiva, efetuada por três avaliadores, com o uso da escala GRBASI. Após testes de confiabilidade intra e interavaliadores chegou-se à classificação final do grau geral do desvio vocal para cada participante. Foram feitas comparações entre grupos que tinham ou não outra profissão/atividade com o uso da voz e os grupos formados a partir do grau geral.

**Resultados:**

A maioria dos estudantes apresentou alto risco para disfonia. Todos apresentaram alteração vocal, com predominância de grau leve. Os estudantes que tinham outra profissão/atividade com uso da voz obtiveram maior pontuação no protocolo específico para atores, bem como na soma desse protocolo com o de rastreio geral. Não houve relação entre o grau de alteração vocal e o risco de disfonia. Os estudantes que ainda não atuavam profissionalmente apresentaram mais alterações vocais de grau moderado ou severo, e os que atuavam profissionalmente apresentaram com maior frequência alteração vocal de grau leve.

**Conclusão:**

A maior parte dos estudantes apresentou alto risco para disfonia. Todos apresentaram alteração vocal, com predomínio de grau leve. Não houve relação entre o risco de disfonia e o grau de alteração vocal.

## INTRODUÇÃO

A voz é um dos elementos relevantes no trabalho fonoaudiológico com o ator, pois abrange o domínio da expressividade, dos recursos vocais, a pesquisa e a compreensão de todos os órgãos envolvidos na produção da voz com o objetivo de atender as necessidades da voz cênica sem fatigá-la^(1,2)^.

A qualidade vocal, a articulação e a pronúncia clara e precisa são necessárias para o ator garantir a inteligibilidade da sua performance. Distorções articulatórias e vocais são toleradas apenas quando atreladas ao processo criativo da cena^(3)^. Na literatura há estudos que demonstraram que os atores são um grupo de risco para o desenvolvimento de distúrbios da voz, com alteração e prejuízo na qualidade vocal^(4,5)^.

A voz do ator também pode ser impactada por fatores como condições ambientais, acústicas e climáticas inadequadas, questões de saúde geral e psicológica^(6)^. Os eventuais problemas de voz do ator surgem pela falta de domínio da ferramenta vocal, das tensões corporais, inclusive no aparelho fonador, da incoordenação pneumofonoarticulatória, e na presença de hábitos inadequados que podem ser observados nessa categoria profissional desde o período de formação^(7,8)^.

A investigação da voz dos alunos, seja em cursos de teatro ou em graduação de Artes Cênicas, é mais escassa^(9)^. No entanto, houve um estudo que verificou a presença de alterações vocais e laríngeas em metade dos estudantes de teatro avaliados^(10)^. Outra pesquisa investigou a prevalência de distúrbios vocais e o conhecimento sobre saúde e alteração vocal durante o período de formação e nos cinco primeiros anos após a graduação. Os atores profissionais citaram maior incidência de alterações vocais nos primeiros cinco anos após a formação e aprenderam menos sobre saúde vocal do que os profissionais do canto erudito e do teatro musical^(11)^.

Para a formação dos atores é importante abordar técnicas de treinamento de voz, aulas sobre saúde e bem-estar vocal, e relacionar os cuidados vocais com a compreensão da anatomia e fisiologia vocal. Reunir amplo e profundo conjunto de informações sobre as características da voz e do estilo de vida desses estudantes pode ajudar os professores de técnica vocal e os fonoaudiólogos que atuam nessa área a usar essas informações como referência para avaliar o comportamento vocal dos alunos, além de identificar quais áreas precisam de mais assistência. Os próprios estudantes podem entender melhor suas condutas e desenvolver a autopercepção vocal como resposta a hábitos e comportamentos^(12)^.

A fim de obter e explorar mais as informações relacionadas aos aspectos vocais, geralmente é utilizada a investigação inicial ou anamnese, pois a pessoa reflete e fornece dados relevantes para compor a avaliação e o planejamento fonoaudiológico^(13)^. Uma forma de realizar essa coleta é por meio do Protocolo de Rastreio de Risco de Disfonia Geral (PRRD-G)^(14)^, um questionário que rastreia o risco de disfonia em indivíduos de qualquer idade e sexo, por meio de análise quantitativa e qualitativa. Esta ferramenta é um instrumento robusto para o rastreio, orientação, prevenção de distúrbios da voz e para auxiliar no planejamento do tratamento. Protocolos específicos complementares ao PRRD-G estão em desenvolvimento, como o Protocolo de Rastreio de Risco de Disfonia para Atores (PRRD-A)^(15)^, com o intuito de investigar fatores do ambiente e organização do trabalho na atuação.

Ao avaliar a qualidade da voz e mensurar o risco de disfonia ampliam-se os conhecimentos sobre as características vocais e obtêm-se informações a respeito do uso da voz na atuação, estilo de vida, saúde vocal e autopercepção vocal dos estudantes de artes cênicas. Tais dados podem contribuir para a criação de ações e estratégias para aprimorar a formação e auxiliar no autoconhecimento por parte desses estudantes, em relação aos seus hábitos, comportamentos e uso vocal, para favorecer a adoção de cuidados e práticas considerando suas rotinas e necessidades.

O objetivo desse estudo foi analisar a relação entre o risco de disfonia e a qualidade vocal em estudantes de graduação em Artes Cênicas. E também comparar o risco de disfonia e a qualidade vocal dos estudantes, que além da atuação, tinham outra profissão ou atividade com uso da voz, com os alunos que exerciam apenas a atuação.

## MÉTODO

### Tipo de estudo

Estudo transversal observacional aprovado pela Comissão de Ética para Análise de Projetos de Pesquisa do Hospital das Clínicas da Faculdade de Medicina da Universidade de São Paulo, sob número (n° 3.315.275), com anuência da instituição coparticipante.

### Participantes

Os estudantes de todos os períodos do Curso Bacharelado em Artes Cênicas de uma instituição de ensino superior, matriculados em 2019, foram convidados para participar da pesquisa. Houve exclusão de indivíduos que apresentassem qualquer comprometimento ou diagnóstico que limitasse a comunicação ou a realização das tarefas propostas no estudo. A amostra foi composta por 38 estudantes, do primeiro ao último período do curso, nos quais foram 11 do sexo masculino e 27 do sexo feminino, com média de idade de 21,3 anos (dp=2,50), mediana de 20,5, variando entre 18 e 29 anos. Todos assinaram o termo de consentimento livre e esclarecido.

### Procedimentos

#### Protocolos

Os participantes preencheram dois instrumentos:

Protocolo de Rastreio de Risco de Disfonia - Geral (PRRD-G)^(14)^, que é composto por questões iniciais de identificação pessoal e mais 18 questões divididas nos seguintes subitens: histórico de disfonia, sinais e sintomas, uso da voz fora do trabalho, alimentação, hidratação, medicamentos, contato com fumantes, sono, histórico de doenças, histórico familiar de distúrbios vocais, atividades físicas e lazer, entre outros. Contempla ainda a autoavaliação vocal, a partir de uma escala analógica visual (EAV), que é medida utilizando régua milimetrada, com valor entre 0 (sem distúrbio) e 10 (distúrbio máximo). Para o cálculo do escore total cada subitem gera uma pontuação parcial, que é somada ao valor obtido na autoavaliação vocal. Essa soma resulta na pontuação total máxima de 131 pontos e, quanto maior o escore final, maior o risco de disfonia. Os escores de 29,25 para mulheres e 22,75 para homens são as referências de cortes para alto risco de disfonia^(14)^.

Protocolo de Rastreio de Risco de Disfonia - Atores (PRRD-A)^(15)^, constituído de 24 questões separadas em subitens, como: formação, aulas de canto, tempo médio de uso da voz, comprometimento vocal, prática de aquecimento e desaquecimento vocal, ensaios/aulas, condições ambientais no uso da voz na atuação, tipo respiratório, psicodinâmica vocal, percepção da voz, atuação em outras profissões ou atividades que utilizam a voz, consumo de fumo, álcool e drogas, uso de prótese dentária e questões voltadas para as mulheres sobre sintomas pré-menstruais, gravidez, menopausa e alterações hormonais. O escore é formado a partir da soma dos pontos de cada subitem. O escore total pode variar de 0 a 100 e, quanto maior o escore, maior o risco de disfonia. Os valores de corte para risco de disfonia deste protocolo ainda estão sendo definidos.

Após a aplicação e mensuração do escore total PRRD-G os participantes foram divididos em dois grupos, segundo a classificação de risco: alto e baixo risco para disfonia a partir do ponto de corte previamente definido pelo protocolo, de acordo com sexo. O escore total do PRRD-A também foi calculado e, com a soma dos totais de ambos, foi gerado o escore final (PRRD-Final). Essa pontuação permite quantificar o risco de disfonia considerando os fatores gerais, aos quais toda população está exposta, e os específicos da profissão, que para o presente estudo foram os agentes relacionados aos fatores pessoais, de organização e do ambiente da atuação.

### Gravação da voz

As amostras de fala foram coletadas com uso de iPad*®* (MP2F2BZ/A, *iOS* 10.3.3), aplicativo *Shure Motiv®* desenvolvido pela *Shure®*, em 44.100 Hz, *monosound* em formato *WAV*, com o microfone unidirecional acoplado, modelo *MOTIV MV88®* (Shure, Estados Unidos), posicionado a uma distância de 15 cm da boca do participante, em ângulo de 45°. Tal medida foi adotada por apresentar melhor qualidade após testes com o microfone em diferentes posições e distâncias. Em um mesmo ambiente controlado acusticamente com ruído abaixo de 50 dB, por meio do aplicativo *SoundMeter*, desenvolvido pela *Digital SoundMeter,* cada um dos participantes foi posicionado sentado para a gravação das seguintes tarefas: emissão sustentada da vogal /a/ durante três a cinco segundos, por três vezes e com *pitch* e *loudness* habituais; fala espontânea a partir da pergunta: “Fale-me da importância da voz para o ator”; e leitura do texto “A descoberta de Cristóvão Colombo” de Mowa Lebesque^(2)^. A pesquisadora segurou o papel com o texto para os participantes lerem e a instrução repassada foi para que primeiro fizessem a leitura silenciosa para se familiarizarem com o texto e, após, realizarem a leitura em tom habitual.

### Avaliação vocal perceptivo-auditiva

A avaliação perceptivo-auditiva da voz foi realizada por três juízes fonoaudiólogos especialistas em voz, com tempo de experiência entre oito e 15 anos, habituados a utilizar a escala de avaliação e com prática em voz profissional. Os registros de voz foram armazenados com 20% de repetição aleatória para análise de confiabilidade intraexaminador.

Cada juiz realizou a análise perceptivo-auditiva das vozes, de forma independente, com o uso da escala GRBASI. Foi solicitado que avaliassem primeiramente a amostra da emissão da vogal /a/ e, após o intervalo de uma semana, para ajudar a reduzir o impacto da memória do avaliador, realizassem a análise da fala espontânea e da leitura do texto. Foram extraídas as cinco frases da primeira metade do texto, que foram lidas por todos os participantes de forma mais fluente. Os examinadores poderiam escutar cada faixa de gravação quantas vezes julgassem necessárias para analisar. Para fins de análise nesta pesquisa foi considerado apenas o G (grau geral do desvio) das avaliações das tarefas vocais.

Com o intuito de calibrar os juízes, foram utilizadas amostras âncoras das características de voz e/ou níveis de grau de desvio. O número e o tipo ideal de âncoras não são conhecidos, então foram utilizados 16 estímulos-âncora da emissão vogal sustentada /a/ e de sentenças do protocolo CAPE-V ou amostras de fala espontânea, contendo quatro amostras de indivíduos vocalmente saudáveis, quatro amostras de indivíduos com desvio vocal leve, quatro amostras de indivíduos com desvio vocal de leve a moderado e quatro amostras de indivíduos com desvio vocal intenso. Em cada grau de desvio vocal havia duas amostras de vozes masculinas e duas amostras de vozes femininas. Os juízes foram orientados a escutar os estímulos-âncora imediatamente antes da análise das vozes desta pesquisa.

### Análise dos dados

A análise descritiva incluiu frequências absolutas e relativas, bem como as respectivas medidas de tendência central e dispersão.

As análises intra e interavaliador da avaliação vocal perceptivo-auditiva foram calculadas com o coeficiente de correlação intraclasse (ICC). O valor de significância estatística adotado foi de 5% (p ≤ 0,05) e foi utilizado intervalo de confiança de 95%. A seguinte classificação foi adotada: valores menores que 0,5 confiabilidade ruim; entre 0,5 e 0,75 moderada; entre 0,75 e 0,90 boa; e maiores que 0,90 excelente^(16)^. Posteriormente às análises de confiabilidade foram utilizados os dados correspondentes aos examinadores com maior índice intra e correlação interavaliador em pelo menos uma das avaliações: vogal/a/, fala espontânea e leitura de texto.

A partir da análise de concordância interavaliadores, os juízes 1 e 2 na tarefa da vogal /a/ apresentaram concordância moderada (0,53; p < 0,001). Na confiabilidade intra-avaliador esses examinadores apresentaram, na mesma tarefa, confiabilidade excelente (1,0; p< 0,001) e moderada (0,71; p < 0,019), respectivamente. Assim, foram selecionadas as análises dos avaliadores 1 e 2 na tarefa de vogal /a/ para a construção da classificação final do G para cada participante. Quando não houve consenso entre os avaliadores foi utilizada a média das avaliações dos mesmos. Então, as amostras foram categorizadas com base na seguinte classificação: 0 a 0,4 - variação normal da qualidade da voz (0); 0,5 a 1,4 - variação leve (1); 1,5 a 2,4 - variação moderada (2); 2,5 a 3,0 - variação severa (3)^(17)^. A partir dessa classificação os participantes foram divididos em dois grupos: grau de alteração vocal leve (grau 1) e grau de alteração vocal moderada e severa (graus 2 e 3).

Para verificação do cumprimento do pressuposto de normalidade e de homocedasticidade foram utilizados os testes de Shapiro-Wilk e de Levene, respectivamente. E puderam ser definidos o teste exato de Fisher (dados categóricos), teste t de Student (dados quantitativos, quando não violado o pressuposto de normalidade) e teste U de Mann-Whitney (dados quantitativos, quando violado o pressuposto de normalidade). Quando violado o pressuposto de heterocedasticidade, o valor de p foi calculado com a correção de Welch. Além disso, foram considerados os coeficientes d (paramétrico) ou r (não paramétrico), nos momentos oportunos, para verificar o tamanho do efeito da diferença entre os grupos. Os critérios adotados para o coeficiente d foram: pequeno: entre |0,200| e |0,499|; médio: entre |0,500| e |0,799|; e grande: acima de |0,800|; e para o coeficiente r foram: pequeno: entre |0,100| e |0,299|; médio: entre |0,300| e |0,500|; e grande: acima de |0,500|.

As seguintes comparações foram realizadas:

- Subitem outra profissão/atividade com uso da voz (OP), grupos que tinham ou não foram comparados em relação: ao escore total do PRRD-G; subitem autoavaliação vocal (EAV); escore total do PRRD-A; PRRD-Final; atuação profissional e orientações vocais (questões do subitem Formação); risco de disfonia;
- Grau de alteração vocal, grupos com alteração leve e alteração moderada/severa foram comparados em relação: ao escore total do PRRD-G; subitem autoavaliação vocal (EAV); escore total do PRRD-A; subitem outra profissão/atividade com uso da voz (OP); atuação profissional e orientações vocais (questões do subitem Formação); PRRD-Final; risco de disfonia.

Nas análises inferenciais o valor de significância estatística adotado foi de 5% (p ≤ 0,05). Para o cálculo dos intervalos de confiança de 95% foi usado o método de viés corrigido e acelerado com base em 2000 amostras *bootstrap*. Foi utilizado o *software* estatístico SPSS^®^ (*Statistical package for the social sciences*) versão 25.0.

## RESULTADOS

Na amostra total observaram-se médias de 37,62 (±8,81) no escore do PRRD-G, 26,18 (±6,93) no PRRD-A e 64,00 (±12,23) no PRRD-Final. Quanto à classificação para o risco de disfonia, 4 participantes (10,5%) apresentaram baixo risco e 34 participantes (89,5%) alto risco.

Sobre a análise perceptivo-auditiva da voz, a partir do parâmetro G, as médias obtidas pelos avaliadores 1 e 2 na tarefa da vogal /a/ foram: 1,37 (±0,49) e 1,16 (±0,79), respectivamente; e nas tarefas de fala espontânea e texto foram: 1,05 (±0,46) e 0,63 (±0,71), respectivamente. Na divisão dos grupos em relação ao grau de alteração vocal, 22 participantes (57,9%) tinham alteração vocal leve e 16 participantes (42,1%) moderada ou severa.

Quanto às comparações, a diferença estatisticamente significativa ocorreu no escore total do PRRD-A e no PRRD-Final, em que os indivíduos que tinham outra profissão ou atividade com uso da voz pontuaram mais do que os indivíduos que não realizavam (Tabela 1).

**Tabela 1:** Valores descritivos e análise comparativa dos indivíduos que realizavam e que não realizavam outra profissão/atividade que usa voz em relação à pontuação nos instrumentos PRRD-G, PRRD-A, PRRD-Final e EAV

**Instrumento**	**Outra profissão/atividade que usa voz**	**n**	**Média**	**DP**	**Mediana**	**Mín.**	**Máx.**	***p***	**T.E.**
PRRD-G	Não	23	36,13 [33,37, 38,94]	7,36	37,00 [33,00, 39,00]	22,00	51,00	0,308^a^	0,408^d^
	Sim	15	39,13 [33,93, 43,87]	10,59	41,00 [37,00, 44,00]	15,00	56,00		
PRRD-A	Não	23	22,91 [20,87, 25,04]	5,05	23,00 [20,00, 25,00]	15,00	34,00	<0,001^a^*	1,640^d^
	Sim	15	31,20 [28,40, 34,27]	6,50	28,00 [26,00, 36,00]	25,00	43,00		
PRRD-Final	Não	23	59,13 [55,89, 62,61]	9,57	57,00 [56,50, 58,00]	37,00	86,00	**0,004^a^** *	1,170^d^
	Sim	15	70,33 [64,20, 75,80]	12,59	73,00 [68,00, 73,00]	40,00	90,00		
EAV	Não	23	4,00 [3,61, 4,41]	1,09	4,00 [4,00, 4,00]	2,00	6,50	0,718^b^	0,061^r^
	Sim	15	3,87 [3,07, 4,60]	1,55	4,00 [3,00, 5,86]	0,00	7,00		

Teste t de Student para amostras independentes (^a^) e Teste U de Mann-Whitney (^b^)

* Valor estatisticamente significativo no nível de 5% (p ≤ 0,05)

d Coeficiente paramétrico para verificar o tamanho do efeito da diferença entre os grupos

r Coeficiente não paramétrico para verificar o tamanho do efeito da diferença entre os grupos

**Legenda:** EAV = Escala analógica visual; DP = Desvio padrão; Mín. = Mínimo; Máx. = Máximo; T.E. = tamanho do efeito

Não houve diferença estatisticamente significativa entre ter ou não ter outra profissão com uso da voz em relação às variáveis analisadas: risco de disfonia, atuação profissional e orientações vocais (Tabela 2).

**Tabela 2:** Comparação da realização ou não de outra profissão/atividade com uso da voz em relação ao risco de disfonia, atuação profissional e orientações vocais

**Variável**	**Conclusão**	**Outra profissão/atividade que usa a voz**						***p***
		Não		Sim		Total		
		n	%	n	%	n	%	
Risco de disfonia	Baixo	3	13,0	1	6,7	4	10,5	> 0,999
	Alto	20	87,0	14	93,3	34	89,5	
Atuação profissional	Não	11	47,9	6	40,0	17	44,7	0,744
	Sim	12	52,1	9	60,0	21	55,3	
Orientações vocais	Não	13	56,5	10	66,7	23	60,5	0,736
	Sim	10	43,5	5	33,3	15	39,5	

Teste exato de Fisher

Os indivíduos com alteração de grau leve e indivíduos com alteração de grau moderado ou severo obtiveram resultados semelhantes na pontuação do PRRD-G, PRRD-A, PRRD-Final e EAV (Tabela 3).

**Tabela 3:** Valores descritivos e análise comparativa dos graus de alteração vocal em relação à pontuação nos instrumentos PRRD-G, PRRD-A, PRRD-Final e EAV

**Instrumento**	**Grau**	**n**	**Média**	**DP**	**Mediana**	**Mín.**	**Máx.**	***p***	**T.E.**
PRRD-G	Leve	22	37,07 [32,82, 41,36]	10,47	37,50 [31,50, 42,75]	15,00	56,00	0,828^a^^w^	0,056^d^
	Moderado ou severo	16	37,66 [34,90, 40,31]	5,98	38,50 [35,00, 40,00]	27,00	47,00		
PRRD-A	Leve	22	25,82 [23,40, 28,36]	6,22	25,00 [24,50, 27,00]	15,00	41,00	0,708^a^	0,140^d^
	Moderado ou severo	16	26,69 [23,06, 30,31]	8,00	25,00 [21,50, 28,00]	15,00	43,00		
PRRD-Final	Leve	22	62,93 [57,52, 68,39]	13,44	64,50 [55,50, 69,00]	37,00	86,00	0,798^b^	0,043^r^
	Moderado ou severo	16	64,41 [60,03, 69,06]	10,19	63,50 [57,50, 66,00]	53,50	90,00		
EAV	Leve	22	3,93 [3,41, 4,45]	1,32	4,00 [4,00, 4,00]	0,00	6,50	0,686^b^	0,068^r^
	Moderado ou severo	16	3,97 [3,44, 4,59]	1,24	4,00 [3,00, 4,00]	2,00	7,00		

Teste t de Student para amostras independentes (^a^) e Teste U de Mann-Whitney (^b^)

d Coeficiente paramétrico para verificar o tamanho do efeito da diferença entre os grupos

r Coeficiente não paramétrico para verificar o tamanho do efeito da diferença entre os grupos

w Correção de Welch para heteroscedasticidade

**Legenda:** EAV=Escala analógica visual; DP= Desvio padrão; Mín.=Mínimo; Máx.=Máximo; T.E.= tamanho do efeito

Observou-se diferença estatisticamente significativa entre o grau de alteração vocal e a atuação profissional. Os estudantes que não atuavam profissionalmente apresentaram mais alteração vocal de grau moderado ou severo, e os que atuavam profissionalmente alteração de grau leve (Tabela 4).

**Tabela 4:** Comparação dos graus de alteração vocal em relação ao risco de disfonia, realização de outra profissão/atividade que usa a voz, atuação profissional e orientações vocais

**Variável**	**Conclusão**	**Grau de alteração vocal**						***p***
		Leve		Moderada ou severa		Total		
		n	%	n	%	n	%	
Risco de disfonia	Baixo	4	18,18	0	0,00	4	10,53	0,124
	Alto	18	81,82	16	100	34	89,47	
Realização de outra profissão ou atividade que usa a voz	Não	14	63,64	9	56,25	23	60,53	0,743
	Sim	8	36,36	7	43,75	15	39,47	
Atuação profissional	Não	6	27,27	11	68,75	17	44,74	**0,020** *
	Sim	16	72,73	5	31,25	21	55,26	
Orientações vocais	Não	12	54,55	11	68,75	23	60,53	0,506
	Sim	10	45,45	5	31,25	15	39,47	

Teste exato de Fisher

* Valor estatisticamente significativo no nível de 5% (p ≤ 0,05)

## DISCUSSÃO

O presente estudo analisou a relação entre o risco de disfonia e a qualidade vocal em estudantes de graduação em Artes Cênicas, pois na literatura há escassez de pesquisas que abordem esses dados de forma conjunta nessa população. Há estudo voltado à exploração das características da voz dos estudantes de artes cênicas^(10)^, assim como relacionado à obtenção de informações de variáveis relevantes à saúde vocal^(13)^.

Sobre os resultados nos protocolos de rastreio de risco de disfonia, no PRRD-G, a partir da investigação dos fatores gerais relacionados à alteração vocal, como saúde, estilo de vida e hábitos, a média obtida foi superior aos valores de corte para alto risco de disfonia. O uso desse instrumento também foi efetivo na identificação e categorização do risco para disfonia na população em geral^(14)^, assim como em atores profissionais^(15)^, atores de teatro musical^(18)^ e professores^(19)^.

No estudo com atores profissionais houve predomínio de alto risco para disfonia^(15)^, o que corrobora com a atual pesquisa com estudantes. Deste modo, é possível constatar que o risco para disfonia pode estar presente nos atores desde o período de formação. Nessa categoria, estudos anteriores encontraram alta prevalência de alterações vocais^(10,20)^. Outra pesquisa utilizou um formulário específico para coleta e registro de informações de estudantes de artes cênicas e verificou relatos de uma série de comportamentos vocais e estilo de vida que podem aumentar o risco de alteração da voz^(13)^.

De forma complementar ao protocolo geral, foram criados instrumentos de rastreio de risco de disfonia específicos, para a população infantil^(21)^ e profissionais da voz, como atores de teatro musical e professores^(18,19)^. Os protocolos específicos para os profissionais da voz têm se mostrado ferramentas úteis para análise qualitativa, uma vez que não diferenciaram profissionais com e sem alteração vocal, pois estão expostos aos mesmos riscos. Entretanto, o uso dos mesmos fornece dados específicos relevantes relacionados à atividade profissional que podem influenciar no risco para disfonia^(15,19)^. Outras propostas de investigação de fatores relacionados à voz do ator foram realizadas, mas não foi encontrado na literatura similaridade quanto à forma como esses aspectos foram apresentados no protocolo utilizado nesta pesquisa^(6,9,22)^.

No que diz respeito à avaliação perceptivo-auditiva da qualidade vocal, apesar de não ter sido foco da presente pesquisa, observou-se que as médias obtidas no grau geral do desvio vocal foram maiores na avaliação da vogal do que nas outras tarefas, considerando as análises dos examinadores selecionados. De maneira geral, a alteração vocal é mais perceptível na emissão da vogal sustentada do que em tarefas de fala encadeada^(23)^.

A partir da classificação final do G, foi verificada presença de alterações vocais em todos os participantes, com predomínio do grau leve, mas chama atenção o fato de que muitos apresentaram grau moderado ou severo. Esses achados não corroboram com outras pesquisas com atores profissionais, amadores ou estudantes de teatro que realizaram a avaliação perceptivo-auditiva e obtiveram médias compatíveis com qualidade vocal sem alteração^(4,15,24,25)^. Para caracterizar melhor as alterações vocais observadas, sugere-se o aprofundamento da análise dos outros aspectos da escala GRBASI. A ampliação da avaliação vocal perceptivo-auditiva, considerando adicionalmente o filtro à fonte glótica, bem como a realização de outras análises, como a acústica, igualmente devem ser consideradas.

A respeito da presença de alteração vocal em toda a amostra, uma hipótese poderia ser em função do aceite em participar daqueles alunos que tivessem percebido a presença de alteração vocal, ainda que na autoavaliação vocal, subitem do protocolo de rastreio de risco de disfonia geral, os estudantes tenham demonstrado não perceber suas vozes como muito alteradas. Além de que, como será discutido posteriormente, não houve relação entre a autoavaliação vocal e o grau de alteração vocal.

Sobre a comparação entre ter outra profissão/atividade com uso da voz ou não, os participantes que as tinham obtiveram maiores pontuações no protocolo PRRD-A, e consequentemente, valores maiores na soma desse protocolo com o PRRD-G, o PRRD-Final. O valor gerado no subescore relacionado a esse uso vocal, além da atuação, possui alta participação na soma do escore total do protocolo. Então, tal resultado demonstrou que esse aspecto foi um fator relevante dentro da pontuação do PRRD-A para diferenciar os estudantes.

Assim como na presente pesquisa, nos estudos com atores é comum observar o uso da voz em outra profissão ou atividade, além da atuação^(6,22)^. Essa prática pode ser explicada porque muitos possuem dificuldade em se manter financeiramente com dedicação exclusiva à atuação, muitas vezes devido à instabilidade do mercado de trabalho e à falta de valorização da profissão. No caso da população pesquisada, por se tratar de estudantes, fica mais evidente a necessidade de realizar outras atividades ou profissões como auxílio financeiro. Além disso, esses estudantes, por estarem inseridos em um ambiente de formação, com disponibilidade de diversas práticas complementares, podem utilizar a voz em outras demandas, principalmente relacionadas ao canto. A partir disso, uma vez que a literatura pontua relação entre a presença de queixa e alta demanda vocal, inclusive a partir do uso da voz em atividades além da atuação, sugere-se destaque e atenção para esse aspecto nos estudos com essa população^(8)^.

Por fim, quanto às comparações a partir do grau de alteração vocal, os estudantes que ainda não atuavam profissionalmente apresentaram mais alterações vocais de grau moderado ou severo, e os que atuavam profissionalmente apresentaram mais alterações de grau leve, tais achados não concordam com a literatura^(9,24)^. O tempo de atuação como profissional não foi explorado neste estudo. A literatura cita média entre cinco e 20 anos de experiência^(4,8,13)^. A atuação profissional, independentemente do tempo, pode ser um fator preventivo para a alteração vocal nesta amostra estudada. Os estudos futuros podem investigar a relação com outros fatores, como a prática de aquecimento e desaquecimento, a realização de treinamento vocal, a presença de determinados hábitos e estilo de vida, entre outros aspectos que podem auxiliar na prevenção de alterações vocais.

Destaca-se também o fato de não ter sido encontrada relação entre o risco de disfonia e o grau de alteração vocal, o que demonstra que o alto risco de disfonia não gerou maior grau de alteração vocal. O mesmo foi verificado em estudos anteriores com atores profissionais^(15)^ e professores^(19)^. Na literatura consultada não foram encontrados estudos que abordassem essa relação com estudantes de teatro/artes cênicas.

Pesquisas com amostras maiores, de atores tanto profissionais quanto estudantes, são necessárias para averiguar melhor os aspectos que podem estar relacionados com a alteração vocal, visando abordagens mais precisas na prevenção e promoção da saúde vocal desses indivíduos. A maioria dos estudos com atores sobre risco de disfonia e qualidade vocal realizaram análises comparativas entre o antes e o depois da *performance* teatral, com achados variados, como melhora, manutenção da qualidade vocal e presença de risco de disfonia^(4,25)^. Em relação aos estudantes, há um estudo que comparou a qualidade vocal, dados de anamnese e autoavaliação de grupos com e sem alteração laríngea, com melhores resultados no segundo grupo^(10)^.

Adicionalmente, não houve relação entre autoavaliação vocal e o grau de alteração vocal, os participantes avaliaram a sua voz de maneira mais positiva do que os juízes fonoaudiólogos. Entretanto, nesta pesquisa deve-se levar em consideração que os juízes avaliaram alguns aspectos da produção da voz, a partir de tarefas específicas, principalmente a emissão de vogal isolada. Os estudantes não possuem o mesmo conhecimento técnico e, ao se auto avaliarem, consideraram como referência o uso vocal de forma mais ampla, no dia a dia.

Na literatura, um estudo também verificou a não concordância entre a autopercepção da qualidade vocal de atores profissionais com a de outras pessoas^(9)^. Os autores referiram que é relevante que o ator seja capaz de identificar com maior precisão alterações em sua voz para agilizar a busca por tratamentos específicos, atenuando o risco de complicações e consequente restrição de possibilidades de uso da voz na atuação.

Neste sentido, desde o período de formação é importante trabalhar a percepção vocal dos atores para auxiliar na identificação de indícios de alterações vocais. Dessa forma, recursos vocais favorecerão a liberdade de interpretação. Além disso, a autoavaliação vocal está associada com a percepção auditiva e estudos têm verificado que a disfonia comportamental pode estar relacionada a distúrbios do processamento auditivo^(26,27)^.

Sobre o fato de não ter ocorrido associação entre orientações vocais e o grau de alteração vocal, na literatura foi verificado que não houve diferenças quanto ao conhecimento vocal de estudantes com e sem queixas vocais^(8)^. Outro estudo também verificou que o conhecimento e a prática do bem-estar vocal não impactaram nas avaliações vocais realizadas com os atores antes e após uma apresentação teatral^(5)^.

Na amostra, havia estudantes que estavam no início do processo de formação, então podiam não ter recebido ainda orientações sobre bem-estar vocal. Mesmo para os estudantes que as receberam, os conhecimentos podiam não ter sido aplicados, pois muitas vezes para o ator é mais importante se apropriar do personagem, do que considerar a saúde vocal^(6)^. Deste modo, além dos conhecimentos sobre bem-estar vocal, é relevante o aprimoramento de abordagens que considerem o aperfeiçoamento da performance corpóreo-vocal-emocional do ator, com manutenção da saúde vocal, especialmente nos cursos de teatro e de graduação em artes cênicas, para o conhecimento e adesão dos atores ainda no processo de formação.

A avaliação da qualidade vocal e a mensuração do risco de disfonia proporcionaram conhecimentos sobre as características vocais, informações a respeito do estilo de vida, comportamentos e aspectos relacionados ao universo da atuação. O uso dos protocolos de rastreio de risco de disfonia pode ter contribuído para a reflexão e autoconhecimento dos estudantes em relação aos seus hábitos e comportamentos, e auxiliado no engajamento e aumento da adesão aos cuidados e adoção de hábitos mais saudáveis, além do uso vocal mais consciente.

Em relação às limitações do estudo, é relevante complementar a avaliação fonoaudiológica e encaminhar os participantes para avaliação otorrinolaringológica, pois a literatura refere que geralmente os atores apresentam alterações laríngeas devido à disfonia comportamental^(28,29)^.

Análises multivariadas e mais aprofundadas de outros dados dos protocolos de rastreio de risco de disfonia, também serão relevantes para serem consideradas em estudos futuros, visando maior detalhamento e compreensão em associação ao que foi encontrado no presente estudo.

A avaliação vocal é um conjunto de procedimentos para identificar e caracterizar o comportamento vocal, a qualidade da voz e os ajustes utilizados na produção da voz. Assim, quanto mais completa, melhor será a compreensão entre a relação do risco de disfonia e a qualidade vocal. A partir das características individuais das vozes de cada aluno/artista, seus hábitos e comportamento vocal, pode ser possível propor um treinamento direcionado para o desenvolvimento e aprimoramento específico de cada voz, com manutenção da saúde e aperfeiçoamento da performance corpóreo-vocal-emocional do ator^(29)^

Ademais, é importante considerar a investigação do processamento auditivo central nesta população, pois dificuldades nas habilidades auditivas interferem no monitoramento adequado da produção vocal, o que pode contribuir para a manutenção de padrões vocais inadequados e influenciar na percepção da modulação da voz^(30)^, fator muito importante para a atividade cênica.

## CONCLUSÃO

Na amostra de estudantes de graduação em artes cênicas desta pesquisa, observou-se que a maioria apresentou alto risco para desenvolver disfonia e todos apresentaram alteração vocal, ainda que com predominância de grau leve.

Os estudantes que tinham outra profissão ou atividade com uso da voz obtiveram maior pontuação no PRRD-A e no PRRD-Final.

Os estudantes que ainda não atuavam profissionalmente apresentaram alterações vocais de grau moderado ou severo com maior frequência, e os que atuavam profissionalmente apresentaram mais alterações de grau leve. Não houve relação entre o grau de alteração vocal e o risco de disfonia.
